# Evolving transdermal therapeutics: a review on self-dissolving polymeric microneedles *via* 3D printing

**DOI:** 10.1039/d5ra05275k

**Published:** 2025-09-12

**Authors:** Geethu Madhusoodanan, Amrita Arup Roy, Tejaswini Kalkundri, Namitha K. Preman, Komal Rana, Deepanjan Datta, Namdev Dhas, Srinivas Mutalik

**Affiliations:** a Department of Pharmaceutics, Manipal College of Pharmaceutical Sciences, Manipal Academy of Higher Education Manipal 576104 Karnataka India ss.mutalik@manipal.edu; b Department of Pharmacognosy, Manipal College of Pharmaceutical Sciences, Manipal Academy of Higher Education Manipal 576104 Karnataka India; c Manipal – Government of Karnataka Bioincubator, Advanced Research Centre, Manipal Academy of Higher Education Manipal 576104 Karnataka India

## Abstract

In recent years, 3D-printed Polymeric Microneedles (PMNs) have been at the forefront of innovations in several biomedical applications, especially in Transdermal drug delivery (TDD) systems. Biocompatible polymers are preferred for their tunable properties that mimic the natural cellular environment, enhancing their clinical suitability. However, their limitations in mechanical strength and stability often require hybridization with synthetic polymers for optimal PMN fabrication. 3D-printed PMNs enable minimally invasive, patient-centric drug delivery, and this review examines diverse microneedle (MN) designs to enhance TDD efficacy, supporting cost-effective clinical translation. This review highlights key aspects like physicochemical properties and their crucial role in additive manufacturing drug delivery systems, which have been underreported. The different sections delve into the challenges of polymeric resin mixes adapted for vat polymerisation and how they can be considered biocompatible, providing detailed insights into the integration potential within future public healthcare frameworks. Furthermore, the review illuminates the clinical outlook, future potential, and strategic directions of PMNs as a pivotal system for TDD, incorporating progress made over the past decade. This review will explore the prospects, benefits, and drawbacks of drug delivery *via* 3D-printed PMN array, addressing key research gaps essential for advancing the industrialization of this cost-effective drug delivery system.

## Abstract 2

Recent advances in 3D-printed PMN microarrays focus on improved durability, drug delivery, release kinetics, design optimization, toxicity assessment, and *in vitro* studies, highlighting their potential for safe and effective transdermal application.

## Graphical abstract

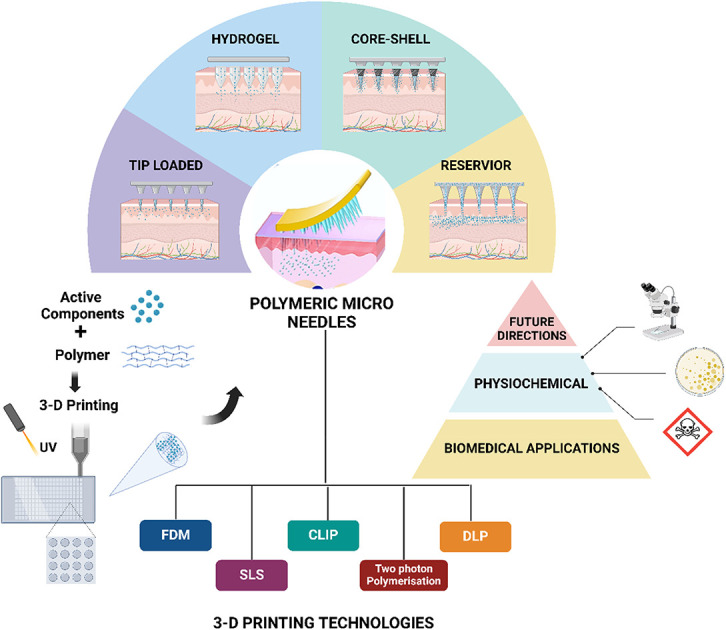

## 1. Introduction

Transdermal Drug Delivery (TDD) systems have significantly contributed to medical practice for the past two decades and cover a wide array of minimally invasive drug delivery systems through the skin. TDD is considered the most promising non-invasive systematic delivery system devoid of hypodermic needles. Focusing on the optimal delivery method and administering active drug components is critical. The regulatory authorities have approved more than twenty effective non-invasive delivery systems to administer active compounds.^1^ The efficiency of a drug delivery system depends on the mechanism and pharmaceutical formulation. Out of the various methods, TDD has emerged as a less risky alternative, bypassing the first-pass metabolism, and overcoming the skin's hydrophobic layer.^2–4^ Recent advancements focus on developing non-invasive, pain-free Microneedle (MN) systems that replace traditional injections and hypodermic needles.

Transdermal MN arrays were developed to combine the benefits of hypodermic needles while avoiding the pain of injection. MN arrays create microchannels in the skin, bypassing the stratum corneum (SC), and enabling drug delivery through passive diffusion. Factors like molecular mass, lipophilicity, and minimum daily dose influence drug permeability. Research shows MN systems effectively overcome challenges in percutaneous drug absorption.^5^ Substantial research outcomes were shown by the MN system, which is an array of MN projections in the range of 50–900 μm height that create transient microchannels that directly help in bypassing the SC layer without stimulating the pain receptors underlying in the tissue layers and actively disperse active pharmaceutical ingredient (API) into the blood by passive diffusion.^6,7^ MN systems have been developed to deliver micro and macromolecules like lipids, proteins, and antigens. These systems are categorized as in-plane, out-of-plane, hollow, solid, swelling, and dissolving, and are made from materials like metals, ceramics, silicon, polymers, and sugar. Among them, PMNs are highly favored due to their cost-effectiveness, easy fabrication, safe disposal, and dissolving properties.^8^

The volume of research in developing PMNs has surged drastically due to its mass production potential, significant cell response, and non-toxicity. PMNs, made from either degradable or non-degradable materials, exhibit varied biological and swelling responses. Their commercial success in advanced drug delivery highlights their superior clinical effectiveness and benefits over other materials, with degradability enhancing bioavailability and sustained drug release.^9,10^ Despite various PMN systems, few have achieved commercial success due to unmet drug formulation standards and persistent challenges. Fabricating PMNs patches involves multiple steps: designing molds, managing spillage, curing, and peeling. Efforts focus on creating less invasive MN with personalized drug release. PMNs are promising for their viability, strength, non-toxicity, cell responsiveness, and stability, but manufacturing techniques greatly influence these qualities.^11,12^ Traditional microfabrication techniques such as micromolding, hot embossing, photolithography, micro-milling, and injection molding, along with first-generation 3D printing approaches like basic fused deposition modeling (FDM) often hinder PMN commercialization due to limited resolution, material constraints, labor intensity, and scale-up challenges. Emerging 3 Dimensional printing (3DP) offers a versatile solution, potentially transforming PMNs manufacturing with its layered approach.^13^

**Fig. 1:**
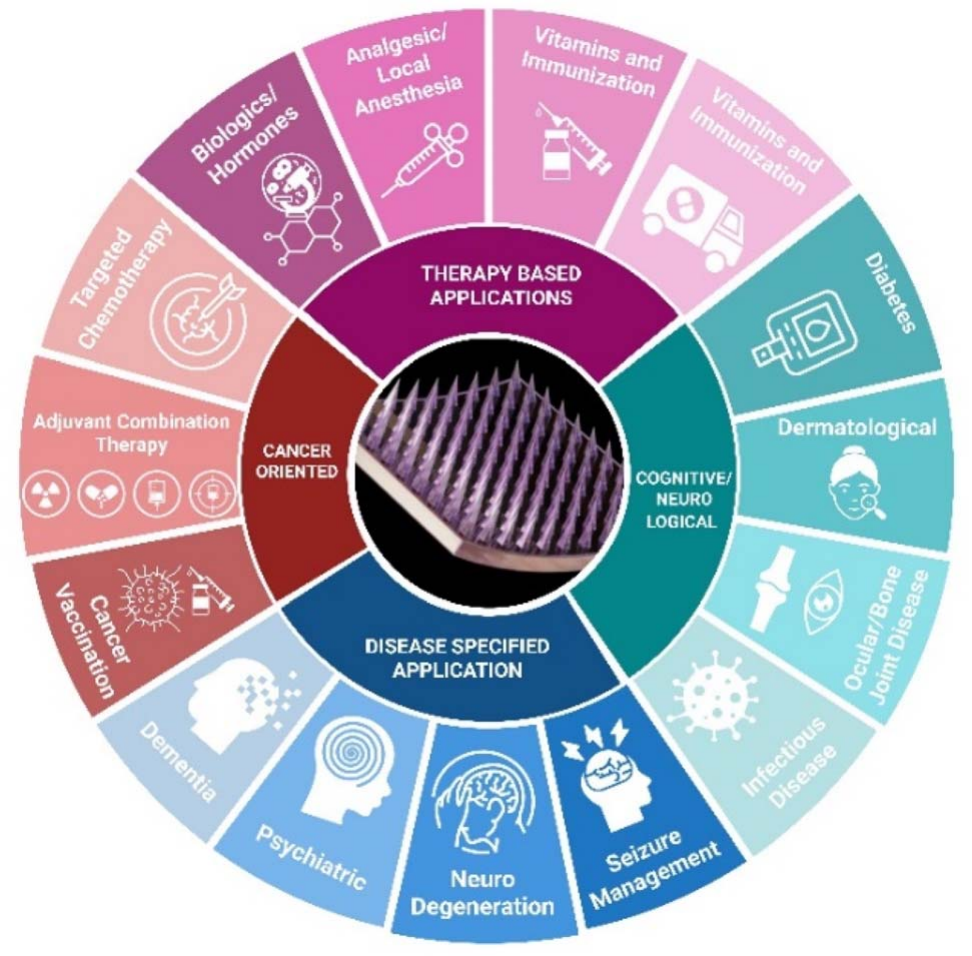
Application of polymeric microneedle (PMN) in pharmaceutical field. Image created using BioRender (http://biorender.com/).

3DP, or additive manufacturing (AM), has become a key method for creating PMNs due to its precision in controlling geometry and composition. In contrast, advanced 3D printing (additive manufacturing, AM) techniques, including high-resolution stereolithography (SLA), selective laser sintering (SLS), inkjet printing, and next-generation FDM systems, build structures layer by layer from digital designs while offering enhanced precision, design flexibility, and rapid iteration. These emerging AM approaches are therefore transforming PMN manufacturing by overcoming the key limitations associated with traditional fabrication techniques. Commonly used biocompatible polymers for 3DP MN include poly (lactic-*co*-glycolic acid) (PLGA), polyethylene glycol (PEG), and polycaprolactone (PCL), which offer mechanical strength, biodegradability, and drug compatibility. Polymeric microneedles (PMNs) have emerged as a promising paradigm in the pharmaceutical industry, offering immense potential. Their multifaceted applications of PMNs are illustrated in Fig. 1.^14^ Advances in MN systems aim to produce simple, less painful, and cost-effective patches, potentially replacing traditional hypodermic needles. However, complex manufacturing processes could slow the commercialization of MN arrays.

**Fig. 2:**
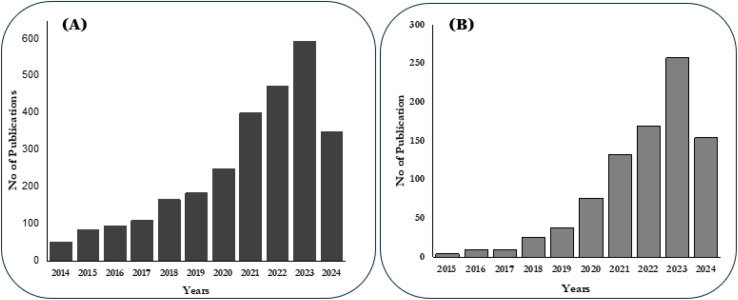
Number of publications on polymeric MN (A) MN-based publications from the past 10 years (2014–2024) using “Polymeric” AND “MN” as keywords (B) 3D printed PMNs-based publications for the past 10 years using keywords “polymeric AND MN AND transdermal AND drug AND delivery” (Source: Scopus).

Over the past decade, the publication rate on PMNs has surged, establishing them as a leading alternative to hypodermic drug delivery. Numerous peer-reviewed articles have explored MN, PMNs, and 3DP techniques. The growing awareness and demand for biodegradable drug delivery systems have significantly boosted the potential of PMNs. Fig. 2 illustrates the rise in publications over the past decade, supporting this trend. However, reviews focusing specifically on 3DP techniques for fabricating PMNs and their drug delivery modes remain scarce. This review addresses recent advances in developing durable PMNs microarray *via* 3DP, including updates on drug delivery systems, release kinetics, design parameters, toxicity, and *in vitro* studies. It will also evaluate the prospects, benefits, and drawbacks of 3DP PMNs for cost-effective drug delivery. Emphasizing TDD using polymeric materials, this review aims to help researchers address early developmental challenges. Additionally, it provides an overview of biomaterial advancements in vat photopolymerization, offering guidance for researchers interested in mold-free MN fabrication and accelerating large-scale commercialization.

### 1.1 Importance of MN delivery system-transdermal method

**Fig. 3:**
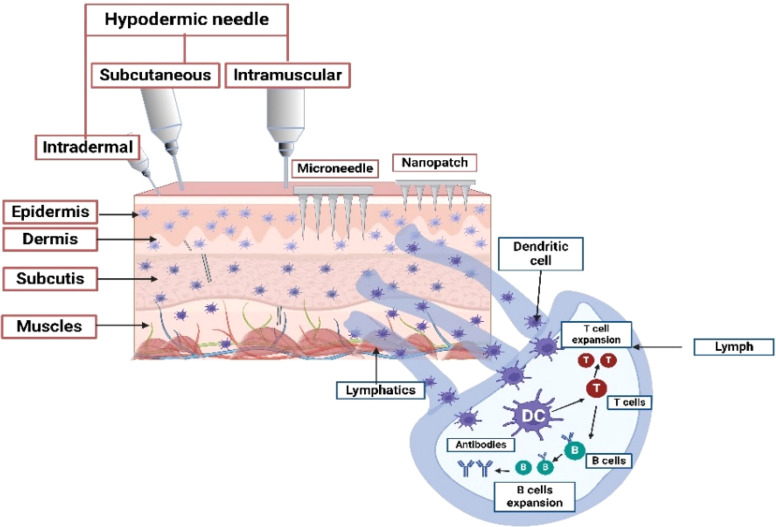
Illustration of comparative needling efficacy of traditional hypodermic needle and MN system in the skin layer. Image created using BioRender (http://biorender.com/).

Hypodermic needles have been the standard for drug delivery for 150 years, but their discomfort has led to the development of pain-free alternatives like MN systems. MN offer advantages such as better delivery, user-friendliness, personalized dosing, and competitive bioavailability. For over a decade, MN-based transdermal delivery has driven innovation in pharmaceutics. MN was carefully tailored to avoid nerve damage and offer a convenient, one-time self-administration option with high drug bioavailability and minimal frequency.^15,16^ Their design and material influence delivery rate and efficiency, accommodating hydrophilic, lipophilic, and amphiphilic drugs with minimal skin damage and extended bioaction. The comparison of the needling and drug delivery efficacy of hypodermic needles and MN is depicted in Fig. 3. This delivery mode maintains the drug plasma levels by spreading into the systemic flow due to consistent infusion. A traditional hypodermic needle typically penetrates deeper into the skin, causing pain, and indirectly reaches the subcutaneous tissue to stimulate lymphatic drainage. MN-based delivery creates superficial microchannels in the skin, which promote targeted delivery into the epidermis, where the dendritic cells transport the APIs to local lymph nodes. This system engages the skin's immune system more efficiently by interacting with the lymphatic drainage. Hypodermic injections are still prevalent, but they don't take advantage of the potential of the lymphatic system for immune stimulation. In contrast, the MN-based system is relatively effective with non-invasive and immune-related treatments.^17^ The extended bioaction period brought about by the increased drug bioavailability reduces the dosing frequency of drugs. The different routes by which the drug enters the systemic circulation also play a vital role in deciding the type of pharmaceutical formulation to be administered through the MN system.^18–20^

## 2. Drug loading strategies using polymeric MN (PMNs)

PMNs have advanced TDD by offering a minimally invasive method to penetrate the SC and deliver drugs directly into the bloodstream. Ranging from tens to hundreds of micrometers in length, they ensure effective, painless administration and improve patient compliance by avoiding the discomfort of hypodermic injections. Additionally, PMNs are versatile, allowing repeated administration and accommodating various therapeutic agents, from small molecules to larger compounds.^8,16^ PMNs provide painless, minimally invasive delivery, enhancing patient compliance by eliminating the fear of hypodermic injections. They also allow for safe disposal, reduce medical waste risks, and preserve sensitive drugs like proteins and vaccines, minimizing cold storage needs. By utilizing various polymers, PMNs enable precise control over drug characteristics and skin performance, ensuring efficient, cost-effective delivery with high encapsulation efficiency. Most self-dissolvable PMNs are made from biodegradable materials like chitosan and PLGA, allowing for controlled drug release and gradual degradation.^10,21–23^

Polymeric solid MN is fabricated in two distinct structural forms: one-piece and layer-by-layer. In the first approach, the solution comprising drugs and polymers is injected and cast simultaneously onto a single mold. On the other hand, the layer-by-layer method involves casting the model structure through successive coating steps or by combining individual flakes of the model.^24^ Different studies were designed and tried to evaluate the capability of such solid biodegradable MN in predicting the drug load capacity, degradation rate, diffusability, and sustained release. PMNs can play a crucial role in releasing drugs in a sustained manner thereby increasing the drug bioavailability by altering the degradation and diffusion curve of the excipients and nanoformulations. Different drug-loading strategies using PMNs can be categorized into four subsections, and they include (i) tip loaded PMNs (ii) drug encapsulated dissolvable PMNs (iii) core–shell filled PMNs (iv) drug reservoir based PMNs.

Drug infusion can occur *via* two main routes: transepidermal and transappendage. Hydrophilic drugs enter through intracellular paths and lipid lamellae of the stratum corneum (SC), while hydrophobic drugs diffuse between SC cells. Large polar drugs, peptides, and proteins are absorbed through hair follicles and subcutaneous glands when delivered *via* microneedle (MN) systems.^25,26^ MNs enable rapid drug action by disrupting the skin, allowing drugs to diffuse into the circulatory system. Drug delivery can be affected by skin physiology, pH, metabolic enzymes, and other factors like anatomical site, skin age, moisture, and body temperature.^27^ The choice of active pharmaceutical ingredient (API) for MN arrays requires careful consideration of factors such as solubility, molecular weight, and irritability, as these influence skin permeation and absorption.^28^

### 2.1 Tip-loaded PMNs

**Fig. 4:**
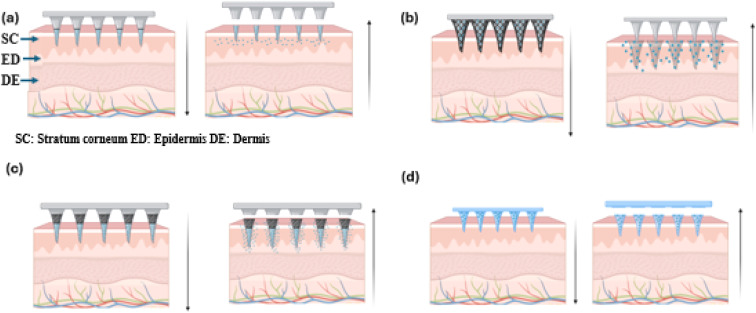
Different MN fabrication strategies used in tip loading solid MN. Image created using BioRender (http://biorender.com/). (a) Tip of solid MN loaded with drug. (b) Bilayer tip with two drugs layered. (c) Tip coated with drug. (d) Drug-loaded MN tip completely inserted into the skin.

PMNs arrays provide versatile options for tailored therapeutic applications through various MN architectures. At their core, MN creates micro-perforations in the skin, allowing for rapid passive diffusion of topically applied drugs into the dermis. More complex designs involve coating drugs on MN surfaces for immediate release upon skin insertion or encapsulating agents within a polymeric matrix, enabling gradual dissolution and drug release into the surrounding tissue. Notably, Park *et al.* (2006) first demonstrated the use of biodegradable PMNs to deliver calcein and BSA in a controlled manner over extended periods.^29^ In 2008, a two-step casting method was introduced for dissolving MN (DMN) that encapsulated lysozyme, BSA, and sulforhodamine B in CMC.^30^ This method has been pictorially represented in Fig. 4a. Advancements in DMN technology led Choi to develop an insertion-responsive MN in 2018, which separated instantly upon skin insertion.^31^ Additional rapid separation methods using air bubbles, effervescence, and high porosity have been explored.^32–34^

Common polymer materials for PMNs include chondroitin sulfate (CS), carboxymethyl cellulose (CMC), polyvinylpyrrolidone (PVP), PLGA, and hyaluronic acid (HA), all designed for skin dissolution with no needle waste.^35,36^ Blending multiple polymers, such as PVP-PVA or PVP-HA, can enhance mechanical properties.^37^ Ideal excipients should be fully degradable to facilitate skin penetration and drug release. Tip-loaded MN systems demonstrate more stable drug delivery than oral routes. For example, studies on solid nanoporous micropile tips using CS for insulin delivery showed sustained, painless release.^38^ Kim *et al.* (2016) successfully delivered high doses of donepezil hydrochloride for Alzheimer's with MN made from HPMC, showing superior efficacy over oral administration.^39^ Wu *et al.* (2021) encapsulated ovalbumin at the tips of PLGA MN, achieving sustained protein release for over two months, and effectively addressing ocular diseases.^40^

The cast drying method for loading MN tips is a simple, efficient process compared to traditional dip and spray coating techniques. It directly deposits drug formulations onto MN tips, minimizing dilution, and wastage, and ensuring higher drug loading efficiency and stability. Unlike traditional methods prone to overspray, cast drying is cost-effective, requires fewer purification steps, and supports various nanoformulations, including solutions, suspensions, and gels. This method is suitable for both immediate and sustained drug release. Gao *et al.* (2019) demonstrated its effectiveness in developing DMN with a gelatin/sucrose film, incorporating BSA, Doxorubicin, and Rhodamine B.^41^ The drug remained stable in the hydrophilic layer, with PEGDA filling the MN tip cavity. Zhou *et al.* (2024) used a similar method for metformin delivery in Type 2 diabetes, achieving stable release for 6–8 hours.^42^ The MN tip with a pre-coated film (Fig. 4b) enhances transdermal delivery, improving skin permeability, compatibility, drug loading, stability, and efficiency compared to dip and spray coating techniques.

The bilayer casting technique, illustrated in Fig. 4c, facilitates simultaneous subcutaneous delivery of multiple drugs. This method, advantageous for releasing dissolvable MN tips into the circulatory system while leaving the drug-free base layer on the skin, relies on polymer dissolution kinetics to control drug delivery timing. Tekko *et al.* (2020) and Peng *et al.* (2021) developed bilayer systems for delivering hydrophilic drugs like methotrexate di sodium (MTX Na) and amphotericin B, using a drug-free base layer to minimize wastage. Their systems featured a solid PMNs tip and a base layer of 20% PVP and 15% PVA, free of drugs.^43,44^ In 2022, Zhang *et al.* created a periodontal dental patch with a dissolvable base infused with tetracycline and MN tips loaded with IL4/TGF-Beta-loaded SiMPs, as shown in Fig. 4d. This method allowed controlled antibiotic release to enhance periodontal tissue regeneration.^45^ However, precise drug concentration loading could compromise the tips' mechanical strength and sharpness. Thus, while the bilayer system effectively loads the desired drug concentration, it may negatively impact mechanical properties.

A major limitation of biodegradable PMNs is their poor skin insertion efficiency, affecting effectiveness. Fabrication typically uses micro-molding with a single master template for all MN, offering precise control but with high startup costs and complex, controlled environment requirements for photolithography and etching.^36^ Modifying designs is challenging with this method, prompting exploration of alternatives like two-photon polymerization and bulk micromachining, though these remain time-consuming and costly for prototype production.^46^

### 2.2 Hydrogel PMNs

Hydrogel-based MN offers promising drug delivery potential by creating microchannels in the skin and accommodating drug reservoirs. The delivery of macromolecules through swellable MN is primarily influenced by the cross-link density of the hydrogel material used in their fabrication.^47,48^ The cross-link density of the hydrogel affects both swelling behavior and the ability to penetrate the SC and encapsulate drugs. Swellable MN, made from cross-linked hydrogels like PEG-PMVE/MA and dextran-PVA, provides the mechanical strength needed for effective transdermal delivery. Various hydrogels such as PEG, PVA-dextran, and other materials like PGA, PLA, PS, PLGA, and chitosan are used for dissolvable MN, which are categorized into dissolvable and swelling systems based on their drug release mechanisms. These hydrogel formulations offer desirable swelling properties and mechanical strength required for effective MN penetration and drug delivery and were preferred for fabricating dissolvable MN for slow release of API.^24,49–51^ Based on the drug release mechanism, they are categorized as dissolvable and swelling MN systems.

**Fig. 5:**
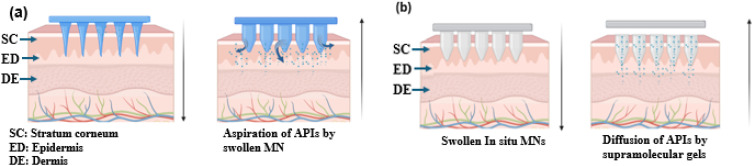
Hydrogel-based drug delivery. Image created using BioRender (http://biorender.com/). (a) Swellable tip delivering drug. (b) Super smart hydrogel delivering drug to skin layer.

Dissolvable MN releases drugs through complete matrix dissolution, ensuring rapid API release and leaving no sharp waste. Techniques like micro-molding, photolithography, and laser ablation fabricate precise PMN arrays for consistent drug delivery.^35^ PMNs are applied by pressing or rubbing onto the skin, allowing MN to penetrate the SC and deliver drugs into the epidermis and dermis by swelling caused due to body fluids, suitable for self-administration (Fig. 5a). Specialized injection devices, like micropillar-based micro-lancers, enable patchless DMN delivery, combining DMN benefits with needle-less jet injectors. Research on insulin-loaded carboxymethyl cellulose (CMC) in micro-lancers achieved 97% efficacy, outperforming traditional DMN.^52^ These devices, with spring-loaded mechanisms or depth control, ensure precise drug delivery and comfort. Intradermal delivery using degradable polymers enhances drug absorption, reduces side effects, and can be further improved with nanoparticle formulations or hydrogel matrices.^53^

Swellable MN enables controlled, sustained drug delivery. Kim *et al.* (2012) developed a PLGA MN system with hydrogel microparticles, allowing simultaneous delivery of hydrophilic and hydrophobic drugs. The combination of hydrophobic PLGA and hydrophilic hydrogel enhanced drug release and reduced insertion time.^47^ Additionally, a biphasic MN ocular patch was designed for treating eye diseases, using HA for rapid IgG release and MeHA for sustained drug delivery. Synergistic transdermal delivery of diclofenac and an antiangiogenic monoclonal antibody was used as API for this study.^54^ Super swelling polymeric materials like Gantrez, PEG, and sodium carbonate combinations were used to fabricate a novel hydrogel-based MN array which was reported to deliver low-potent drugs with clinical relevance.^55^ An attractive feature of hydrogel is the phase transition property which preserves the benefits like improved drug payload and efficient permeation with minimal fabrication steps. In a few hydrogels, the *in situ* swellable nature can be triggered by external stimuli or by physical interaction, usually referred to as supramolecular gels that hold toxin-free crosslinking features making it a versatile drug delivery system and smart gelling mechanism to deliver drug is represented in Fig. 5b.^56^

Dissolvable MN for TDD uses polymers with high swelling capacity, encapsulating the API in a gel matrix that degrades upon skin insertion, releasing drugs. The “poke and release” mechanism expel drugs through channels created during insertion or at the MN tips in the transdermal layer. Key factors include polymer dissolution rate, MN penetration efficiency, drug release kinetics, and transdermal absorption. Hydrogel MN enables macromolecule penetration by forming reversible microchannels (Fig. 5b). Courtenay *et al.* (2018) found dissolving MN provided a higher *C*_max_ (488.7 ng mL^−1^ at 6 hours) for bevacizumab than hydrogel MN (81.2 ng mL^−1^ at 48 hours for 5 mg dose, 358.2 ng mL^−1^ for 10 mg dose). PVA-based DMN allowed immediate release, while hydrogel MN offered sustained release, enabling customization of pharmacokinetic profiles for macromolecules.^57^

DMN arrays for amphiphilic vaccines are favored for their simplicity and safety. Poly acrylic acid (PAA) effectively delivers macromolecules by forming intricate mesh networks. MN loaded with peptide antigens and amphiphilic CpG adjuvants enables self-administration of vaccines.^58^ In 2015, a two-stage process adjusted the PVP–PVA ratio to control drug diffusion in DMN, with oxygen plasma treatment used to fabricate PVP–PVA MN patches for model drugs like rhodamine and fluorescent BSA.^59^ In 2022, a nano-emulsion-loaded DMN array using PVP–PVA facilitated rapid polymer dissolution for lipophilic drug delivery, showing promise for improving amphotericin B administration, particularly in dermatological applications.^60^ Further research and clinical trials are needed to confirm the efficacy and safety of MN for clinical use.

### 2.3 Core–shell filled PMNs

Core–shell-filled PMNs represent a promising drug delivery system, encapsulating drug-loaded cores within dissolvable or biodegradable needles. This versatile approach allows customization for delivering various therapeutics, including small molecules, biologics, vaccines, and nanoparticles. Researchers can tailor the core properties to achieve specific release profiles, targeting capabilities, and drug-loading capacities, making it suitable for diverse applications in drug delivery and biomedical research. Different fabrication methods are employed to create filled MN tips, either using hollow MN molds or directly mixing nano-formulations into the tip spaces. A common technique is coating, where drug-loaded cores are covered with a dissolvable or biodegradable shell using methods like dip coating, spray coating, or electrostatic deposition, followed by integration into the MN structure.

**Fig. 6:**
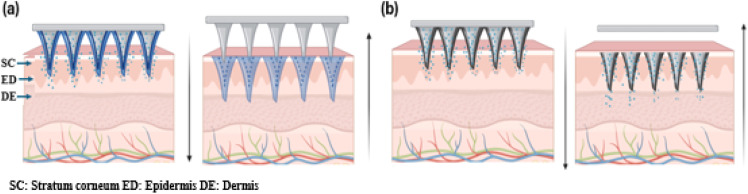
Two methods of drug delivery mediated through core–shell filled MN. Image created using BioRender (http://biorender.com/). (a) Three-layered core–shell MN loaded with different drugs. (b) Drug embedded in the core of MN with a delaminated polymeric outer layer.

In the emulsion or solvent casting method, drug-loaded cores are encapsulated in a polymeric shell, allowing precise control over the composition and properties. API protected within the core–shell enhances the stability and shelf life, as the shell acts as a barrier and protects the drug payload from environmental factors. This core–shell structure enhances the stability and shelf life of the API, protecting it from environmental factors. Li *et al.* developed a sequential casting method to encapsulate the contraceptive hormone levonorgestrel (LNG) in a monolithic PLGA core with a PLLA shell and a PLA backing. This design enables controlled release of LNG for months, reducing patch applications to twice a year, while minimizing burst release and maintaining API potency.^61^ The core shell filled with drugs was pictorially represented in Fig. 6. Similarly, nano-emulsions of estradiol valerate (E2V) were encapsulated in chitosan-based dissolving CS-DMN for transdermal estrogen replacement therapy, achieving sustained release. PCL was used as the core polymer, while Gantrez S-97 formed the backing layer. CS-MN patches have also been used for oral ulcer treatment, incorporating dexamethasone and zeolite imidazoline, and another design with verteporfin and heparin for scarless healing. Lyu *et al.* (2024) developed a core–shell MN with a GelMa outer shell containing mangiferin and a PGLA-DMA core with exosomes for advanced tissue regeneration.^62–64^ A versatile three-step casting method represented in Fig. 6a allows the scalable fabrication of MN patches using various materials, including polymers, hydrogels, and nano-formulations, ensuring consistent quality. This method enables customization for both immediate and prolonged drug release, as demonstrated by two recent studies. In 2024, researchers developed self-dissolvable PMNs for hyperuricemia treatment, using a CMC outer shell with allopurinol and a PVP-urate oxidase core, effectively reducing serum uric acid levels. In 2018, Wang *et al.* introduced a similar layered dissolvable MN with a HA shell and PVP core, offering high drug delivery efficiency and stability. The three-step casting method allows for precise drug formulation but faces challenges like layer adhesion and delamination.^62,64^ This sequential layer casting method allows for precise customization of drug formulations, improving stability and minimizing leakage and the layered MN are pictorially represented in Fig. 6b. However, challenges such as layer adhesion and delamination need to be addressed to avoid premature degradation, which complicates polymer selection.

Dissolvable core–shell MN enable targeted drug delivery with site-specific release and reduced side effects. However, complex fabrication increases costs and risks, such as core material leakage, which can impact drug efficacy. Risks such as core material leakage during fabrication or application can lead to inconsistent drug delivery and loss of efficacy. Focusing on optimizing drug release in early studies could provide economic benefits and improve TDD for better patient-centric therapeutic solutions.

### 2.4 Drug reservoir-based PMNs

Reservoir-based PMNs patches represent an innovative approach to drug delivery, integrating drug reservoirs into the MN base or backing layer. This design facilitates slow drug penetration through micro-orifices, enabling controlled and precise drug release. A key advantage is the ability to achieve specific release kinetics, including pulsatile delivery tailored to therapeutic needs. This helps maintain stable drug levels, enhancing patient compliance and minimizing dosing frequency. Additionally, these patches improve drug stability and shelf-life by protecting against environmental factors, and ensuring long-term efficacy.^16^

**Fig. 7:**
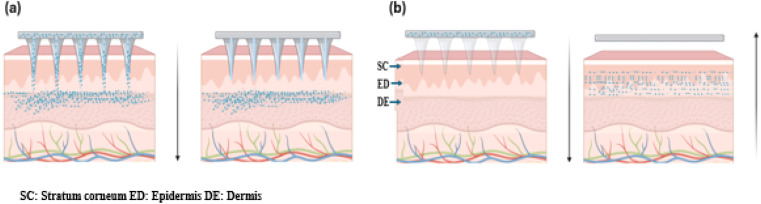
Drug delivery strategy mediated through reservoir-based MN system. Image created using BioRender (http://biorender.com/). (a) Drug loaded in the reservoir diffusing through the conduits. (b) Reservoir drugs get completely diffused through dissolvable MN.

The reservoir-based MN delivery system allows for precise control over drug release rates. Early designs utilized biocompatible polymeric materials, such as polyglycolide acid (PGA), to create 300 μm-long reservoirs for blood extraction without clogging. A notable advancement was an out-of-plane hollow MN with cylindrical side openings, integrating a glass chamber reservoir for transdermal insulin delivery, demonstrated through simulation-based designs.^65,66^ Another study utilized biocompatible silicon carbide (SiC) to develop MN with valves for improved drug release control. These MN enable TDD *via* interstitial fluids, with the drug moving from the reservoir through swollen MN to the skin and dermal microcirculation As represented in Fig. 7a. Common materials for hydrogel MN reservoir patches include poly (methyl vinyl ether-*co*-maleic acid), Gantrez, chitosan, PLGA, and PVA.^67,68^

A novel patch-type drug reservoir attached to hydrogel-forming MN creates continuous conduits upon skin insertion, enabling sustained drug delivery controlled by the MN's crosslinking density. This system, designed for high-dose drugs using hygroscopic lyophilized reservoirs, allows drug permeation through interstitial fluids into the dermal microcirculation is pictorially represented in Fig. 7a. Common materials for these hydrogel MN include poly (methyl vinyl ether-*co*-maleic acid), Gantrez, chitosan, PLGA, and PVA.^69^ Recent advancements feature super-swelling DMN combined with wafer-type drug reservoirs, effectively delivering large molecular-weight drugs. One study demonstrated this method by delivering ibuprofen sodium and ovalbumin, achieving 49% delivery efficiency over 24 hours. Similar research explored delivering hydrophobic drugs like olanzapine and atorvastatin using co-solvent-based reservoirs.^55^ Lyophilized wafers have also been employed for metformin, esketamine, and bevacizumab delivery.

Additionally, a nanoporous MN array with an external liquid reservoir has been developed to assess the diffusion of low molecular weight drugs.^70^ While these reservoir formulations show promise, interactions between drugs and matrix polymers can affect drug stability and release. These works were proof that the manipulation of MN reservoirs is imperative to expand the realm of hydrogel MN beyond the solubility of drugs. A nanoporous MN array paired with an external liquid drug reservoir (Memetine) was developed to study the diffusion of low molecular-weight drugs and represented in Fig. 6b. This *in silico* model effectively predicted the relationship between drug release profiles, concentration, and reservoir volume.^71^ However, these reservoir formulations may not suit all drugs, as interactions between the drug and matrix polymers can affect stability and hinder release.

Polymeric films and lyophilized wafers used in reservoir systems for MN arrays are primarily water-based, requiring stable drug molecules in an aqueous environment. However, unstable drugs need alternative reservoir solutions to prevent degradation. A study explored a nonaqueous reservoir-based MN array with a directly compressed tablet (DCT) system, testing various low molecular weight drugs like amoxicillin, atenolol, primaquine and levodopa. This solid-state reservoir ensured stability for drugs that degrade in water. *In vivo* results demonstrated that these novel MN patches can enhance the transdermal bioavailability of drugs that typically cannot penetrate the skin effectively.^72^

A synergistic approach that capitalizes on the use of two dry drug reservoirs, the DCT and lyophilized wafer (LYO) method along with hydrogel was reported by different groups in 2022. The same year, researchers introduced a synergistic approach combining directly compressed tablets (DCT) and lyophilized wafers (LYO) with hydrogel for TDD. This method enabled the delivery of cefazolin (CFZ), a highly polar drug, at a high concentration (1.5 mg), using a Gantrez S97-carbopol-based hydrogel-forming MN patch for localized infections. The combination of the tablet and wafer reservoirs increased overall drug payload and maintained the stability of nonpolar drugs over time with controlled release. Additionally, a hybrid MN patch with a porous gel drug reservoir was developed for self-regulated insulin delivery, featuring a PVA-coated boronate MN patch for improved skin penetration.^73^ The overall drug payload was reported to increase with compressed tablet and wafer dry drug reservoir system. The stability of the nonpolar drug in the lyophilized wafer helped to ensure the integrity over a long time with precise control over the drug release profile.^74^

## 3. 3D printing in the fabrication of PMNs

**Fig. 8:**
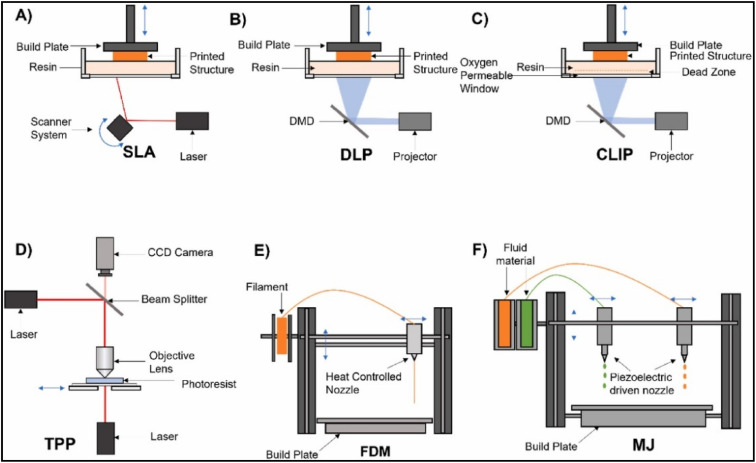
Illustration of different 3D fabrication processes and types (reproduced from J. M. Loh *et al.*,^75^ with permission from Elsevier B.V, © 2023). (A) Stereolithography (SLA); (B) digital light processing (DLP); (C) continuous liquid interface production (CLIP); (D) two-photon polymerization (TPP); (E) fused deposition modelling (FDM); (F) material jetting (MJ).

Recent advancements in scientific and technological techniques have greatly improved the rapid production of versatile MN. Traditional methods for fabricating polymeric MN are valued for their high replicability, scalability, and cost-effectiveness. However, they face limitations due to complex processes, costly equipment, and struggles with non-planar surfaces, making it challenging to achieve all desired features simultaneously. Recently, 3DP has significantly evolved, simplifying manufacturing with its tunable and flexible approach, greatly enhancing fabrication.^8^ which is a viable alternative for MN systems, enabling the easy creation of customizable, intricate polymeric structures. Various 3DP techniques for MN are shown in Fig. 8.

This 3DP technique is a recent advancement in MN fabrication, enabling the creation of complex, precise structures with customizable geometries tailored to individual patient needs. This technology is used to create MN for drug delivery, vaccination, and other medical applications by designing them with software and printing them in layers using biocompatible materials.^76,77^ A key advantage of 3DP for MN is its ability to create complex structures, like multi-branched or varying needle lengths, that are hard to achieve with other methods. 3DP uses versatile biocompatible polymers to match desired properties and drug release profiles. Once designed, it can be scaled for efficient large or small-scale MN production, with minimal waste. This technique uses versatile biocompatible polymers to match desired properties and drug release profiles. It can be scaled for efficient MN production, from small to large scale, with minimal waste. Recently, researchers have used various techniques to create 3DP PMNs, focusing on functionalities such as drug type, concentration, biodistribution, and material properties. Environmental factors and hygroscopic materials are key in PMN development, as moisture absorption can weaken MN structures and affect their performance. MN production facilities must maintain optimal environmental conditions to achieve geometrical consistency, mechanical strength, and micron-scale precision for effective needle penetration into the skin.^78^

Polymeric MN is conventionally fabricated using traditional indirect method which relay on moulding to cast PMNS which is labour intensive and less precise. With the advances in the 3D printing technology, PMNS fabrication has categorized as indirect and direct additive fabrication approaches. The common 3DP techniques like Stereolithography (SLA), and digital light processing (DLP) are used to make high resolution master moulds which will be later used to make mother moulds using silicon or PDMS, on to which the PMNS will be casted and replicated.^79^ There are different methods to cast polymer blend including polymer melt which offers cost effective simple replication process assuring rapid prototyping. The high temperature used for melting the polymer paved way to the introduction of micro moulding based on solvent casting wherein aqueous polymer blends gets dried in room temperature.^79^

**Fig. 9:**
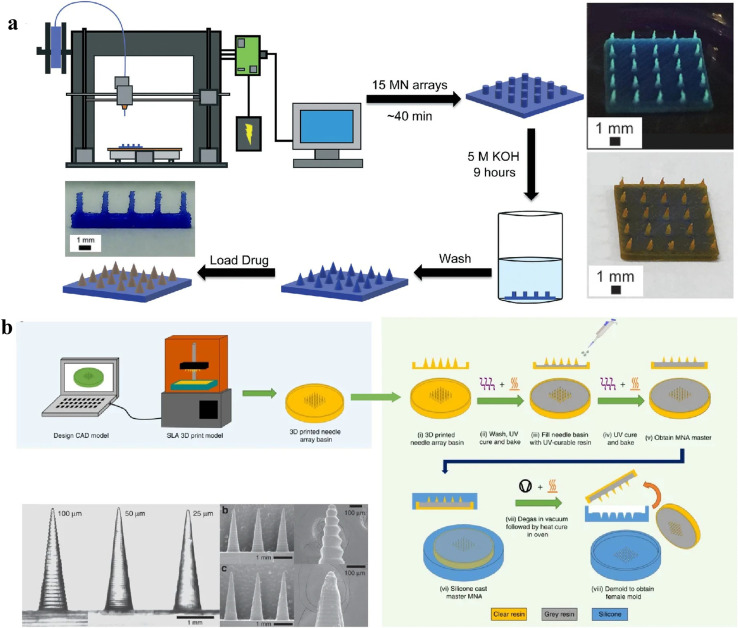
Schematic representation of 3D-printed microneedle fabrication: (a) direct printing of polymeric microneedles using photopolymerization techniques, and (b) indirect method where 3D-printed master moulds used to cast polymeric microneedles (reproduced from X. Luo *et al.*,^81^ with permission from Springer Nature, © 2023).

**Fig. 10:**
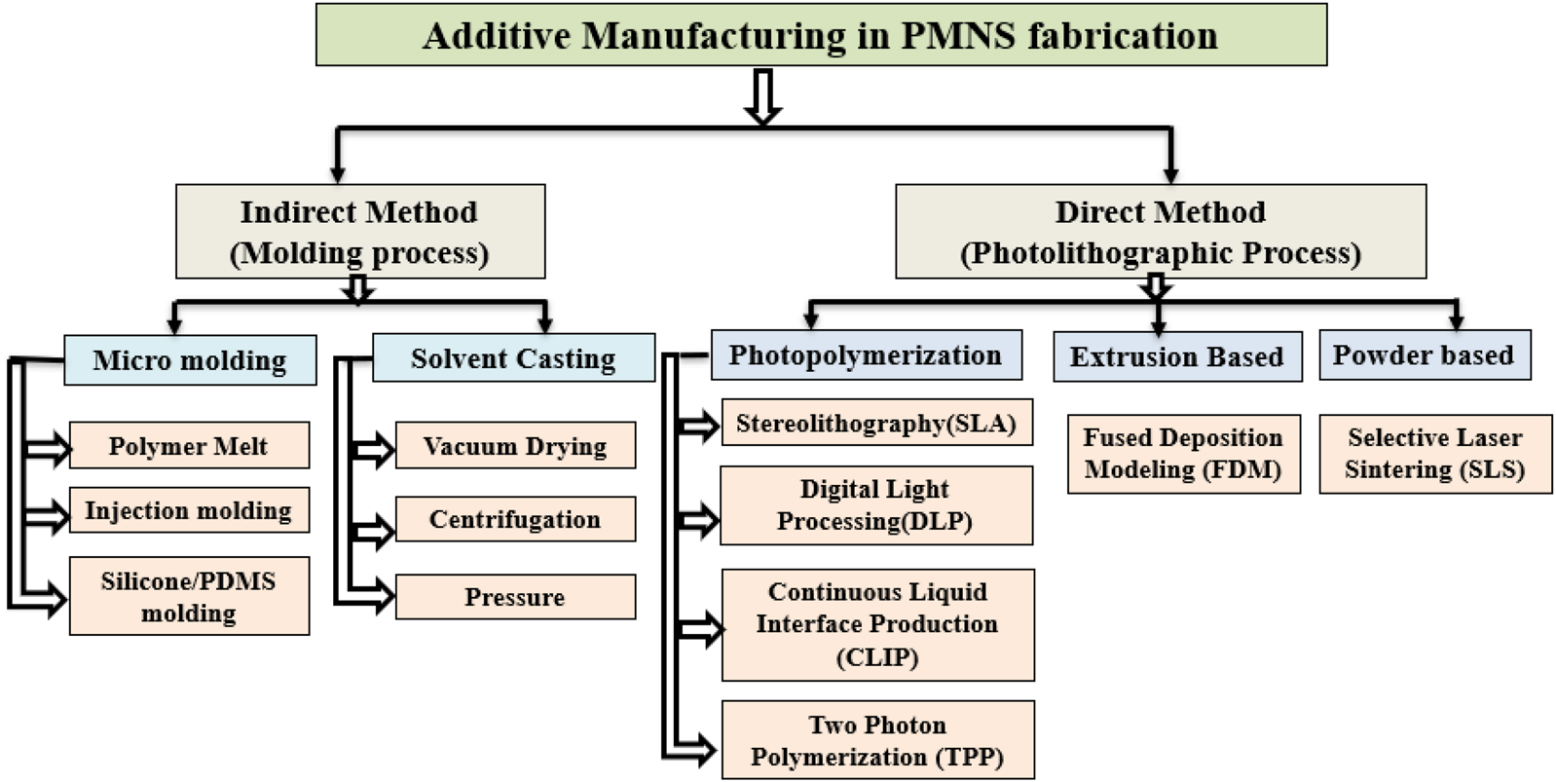
Horizontal flowchart illustrating the two primary approaches for fabricating 3D-printed PMNs.

The limitations of conventional micro moulding techniques have been mitigated through the use of centrifugation and pressure-assisted drying or degassing, which reduce porosity and produce higher-quality microneedles with improved structural integrity. In direct method of fabricating PMNS, photolithography is employed to create MNs in a single step by transferring patterns onto a photoresist, enabling complex and customisable MN geometries MN with sharp precise tips. The steps in the fabrication of 3D printed PMNS is depicted in Fig. 9, which showcases the direct and indirect methods used in printing. For rapid and cost-effective production, 3DP methods such as SLA, DLP, 2PP, and FDM are popular.^36,80^ To clearly illustrate the two primary approaches for fabricating 3D-printed PMNs, direct 3D printing, which fabricates the MNs layer by layer using photopolymerizable resins, and indirect 3D printing, where high-resolution 3D-printed masters are used to create moulds for PMNs casting, are summarized in Fig. 10 as a horizontal flowchart highlighting key techniques and features, providing a clear, visual guide to the 3D printing fabrication process for PMNs.

SLA and DLP systems efficiently produce PMNs but face challenges with resin preparation and MN detachment from the printing base. Though costly, 2PP provides design flexibility for MN. Photopolymerization is advantageous for precise small-scale production, using specific light sources and initiators to solidify liquid resin into desired shapes. Unlike FDM, which uses filament extrusion in a heated environment, photopolymerization relies on controlled light exposure. Traditional manufacturing techniques face limitations like the need for advanced facilities, time-consuming processes, expensive equipment, and limited customizability in parameters such as array size, height, and aspect ratio. AM processes can address these limitations. This section reviews AM techniques for fabricating customizable, complex, and cost-effective polymeric micro-scale 3D structures for drug delivery.

### 3.1 Fused deposition modelling (FDM) technique

3DP technologies offer distinct benefits, making them suitable for various applications in the development of PMNS. FDM stands out as the most economical and widely adopted 3DP method. This technique involves melting a thermoplastic polymer filament at the print head, which is then extruded and deposited layer by layer at the print station while the print head moves by the 2D slice pattern. The sequential buildup of these layers vertically creates a 3D structure, where each new layer fuses with the preceding ones upon cooling below the polymer's melting point. FDM printing operates in a heated environment for polymer filament extrusion. Despite its speed and cost benefits, it has resolution limitations due to its layer-by-layer approach with a large nozzle (400 μm), leading to surface roughness and larger MN sizes.

FDM printing is commonly used for fabricating solid PMNs due to its compatibility with various thermoplastic polymers like PLA, ABS, and nylon. However, FDM-produced objects often have rough surfaces and lack dimensional precision, requiring additional post-processing for smoothing. FDM-printed objects usually exhibit uneven strength and anisotropy, making it difficult to achieve fine details in MN, especially with PLA due to poor layer adhesion. To address this, cylindrical shapes are printed and then chemically etched with KOH to create MN with 1–55 μm tip diameters.^82,83^ However, achieving uniformity remains a challenge, impacting the performance and drug loading accuracy of the final patches. Wu *et al.* developed a 3DP MN system using FDM technology and a post-stretch method by TDD for type 1 diabetes. The MN patches featured tapered needles (6 × 6, base diameter 601 μm, end diameter 24 μm, height 643 μm) designed for controlled insulin release. This minimally invasive system regulated blood glucose in diabetic mice for up to 40 hours, showing promise for sustained TDD.^84^ Song *et al.* also used FDM 3DP for a cost-effective MN system, using elongating PLA filaments to create conoid and neiloid-shaped MN with smooth surfaces. The conoid MN demonstrated effective skin penetration, highlighting the potential of 3DP in painless drug delivery.^82^

Recent publications on FDM-based PMNs in pharmaceutical applications highlight limitations, particularly when drug-loaded polymer filaments are used. In this technique, molten polymer mixed with drugs is extruded through a heated nozzle layer by layer. A key limitation is optimizing the polymer's rheological properties, such as melt viscosity, elasticity, and flexibility, to ensure good thermal stability for effective FDM printing.^22,85^ Most commonly used pharmaceutical-grade polymer lacks the attributes mentioned above either having a high rate of brittleness or very soft gels that cannot be pushed due to the pliability of filaments. Recently, attention has shifted to non-pharmaceutical materials like PVA or PLA, where drugs are mixed *via* solvent diffusion or by combining polymer filaments and drugs for simultaneous extrusion. However, the semi-crystalline structure and high transition temperature of these polymers can affect polymer-drug miscibility.^86^ Using hot melt extruders with plasticizers can degrade APIs, and drugs embedded in MN made *via* FDM often show slow, incomplete release. Thorough evaluation of drug–polymer interactions and miscibility is essential, and screening suitable polymers is crucial for determining commercial viability.

Despite its limitations, FDM 3DP remains widely accepted for cost-effective rapid prototyping with renewable, biocompatible materials. In 2018, Luzurianga *et al.* presented an ideal PLA MN prototype using FDM with a post-fabrication etching step. This group tackled the challenge of creating finer MN structures by improving the KOH-mediated etching step. The model produced finer needle sizes and shapes capable of penetrating the skin and delivering drugs without a master template, enabling the use of less common biocompatible polymers like polyglycolic acid, PLGA, and polycaprolactone, which are not typically compatible with other AM fabrication techniques.^18,86^ Researchers developed a rapid and reliable method for manufacturing MN molds using 3D FDM printing, highlighting advantages like faster production, reduced material use, and the use of Generally Recognized As Safe (GRAS) poly(lactic acid) without needing master templates. The study also showed potential for creating diverse polymeric MN geometries to enhance the TDD of galantamine and other APIs.^22^

### 3.2 Vat photopolymerization (VPP) technique

VPP is one of the most researched and advanced 3DP technologies currently existing. The main advantage is that this technique contributes to the precision and finer fabrication details with high printing speed, which adds to the commercialization aspect of MN technology. Rapid advancement and research based on 3DP have revolutionized nearly all fields of industry, including pharma and medicine. This technique uses photo-sensitive liquid resin as the printing material and there are two primary VPP printing technologies based on curing and way of elevation, they are SLA and DLP.^45,87^ These two primary categories of printing technology stand as the most used method for fabricating transdermal MN. Recent advancements in additive manufacturing have paved the way to develop high-resolution alternatives to DLP systems like continuous liquid interface production (CLIP). Two-photon photopolymerization (TPP) is another cutting-edge method that uses.^88^ Despite the advantages of this technique, a significant challenge is the incompatibility of standard VPP resin mixtures, as most are not biocompatible, raising concerns. This section will discuss the current state of polymeric MN fabrication based on VTT and the novel biomaterials used in transdermal applications in the pharmaceutical and medical fields.

#### 3.2.1 Stereolithography (SLA)

SLA, known for its precision, accuracy, and smooth finish, is widely used for fabricating MN. It features a printing platform with an open or closed resin tank, depending on the application.^89^ SLA relies on the photopolymerization of resin with photoactive monomers, using a UV laser to solidify layers. After printing, the MN is washed with alcohol to remove residues and cured in a UV chamber. While SLA offers fine resolution (∼10 μm), it is slow, expensive, and limited in biocompatible materials. Sourcing suitable light-sensitive, biocompatible materials remains a challenge despite their advantages. This method is widely used to design finer polymeric transdermal MN, but some materials in this technology may pose toxicity risks.^90^

A study developed self-dissolvable PMNs for transdermal insulin delivery using SLA. Insulin was ink-printed onto biocompatible conical MN with stabilizers, and diffusion tests showed rapid insulin release within 30 minutes through porcine skin, highlighting SLA's effectiveness for scalable, biocompatible MN production.^91^ Moreover, SLA has been employed to fabricate hollow microfluidic MN in a single step, demonstrating potential applications in hydrodynamic mixing and combination therapy for preclinical biological therapies.^92,93^ In a recent development, Uddin *et al.*in 2020 utilized SLA to create 3DP PMN arrays delivering cisplatin to epidermoid skin tumors. These MN exhibited strong mechanical properties and excellent penetration depth, as confirmed by optical coherence tomography.

SLA has successfully fabricated various MN types, including solid and hollow variants, using Class 1 biocompatible resins known for their strength. These MN were later coated with insulin-sugar films. Yadav *et al.* demonstrated the fabrication of hollow MN *via* SLA, achieving high fidelity and mechanical robustness suitable for efficient rifampicin delivery through porcine skin.^94^ A hollow MN device with a microfluidic system was fabricated using an SLA printer, allowing for lower cost and faster production. This method supports new TDD approaches for preclinical testing *via* a programmable drug delivery system. The team developed a methacrylic acid ester and PI mix to enable homogeneous drug mixing in the integrated hollow MN microfluidic device. Choo *et al.* optimized the MN design by adjusting SLA printing angles, achieving sharper tips and enhanced structural integrity.^90^ Another study explored varying 3DP angles and aspect ratios to optimize MN platform dimensions, concluding on optimal parameters for needle tip, base diameters, and heights.^95^

Low-cost 3D printers struggle with producing precise MN due to limited mechanical strength and sharpness. To overcome this, Kreiger *et al.* introduced a two-step “Print & Fill” method using SLA 3DP to create customizable MN master molds, evaluated for accuracy and reproducibility.^96^ Yang *et al.* demonstrated SLA's potential in designing morphologically optimized MN with enhanced TDD efficiency, emphasizing mechanical strength and programmable drug release profiles.^97^ Turner *et al.* used SLA for rapid, cost-effective MN templates, which were later used in micro-molding to produce hydrogel MN, achieving performance similar to commercial versions in porcine skin penetration.^98^ SLA also enables the fabrication of solid and hydrogel-based MN with various drug loads, but challenges remain, including a limited material library and stringent quality control.^99^ Volumetric 3DP, a recent development, shows potential for faster PMNs fabrication, but issues like dimensional stability, material compatibility, and post-processing optimization need resolution.

#### 3.2.2 Digital light processing (DLP)

DLP is an efficient printing technique that converts an image signal into a digital signal, which cures light-sensitive liquid resin through continuous vertical movement of the build platform. This method allows for precise, high-resolution object production, reducing the need for extensive post-processing and enhancing production efficiency. Compared to other strategies, DLP achieves outstanding smooth surfaces and rapid production of micrometer-scale MN by curing resin layer by layer using digital light projectors. DLP produces PMNs with consistent geometries, essential for efficient skin penetration and effective drug delivery. Gittard *et al.* (2011) were the first to use DLP to create MN of various geometries for wound healing applications, employing liquid acrylate-based resin through visible light dynamic mask micro-stereolithography for rapid prototyping. They subsequently used pulsed laser deposition to apply thin films of silver and zinc oxide to MN arrays. Light-based technologies have proven valuable in fabricating antimicrobial MN patches.^100^ Lu *et al.* (2015) used DLP photopolymerization to create MN arrays from poly (propylene fumarate)/drug blends, incorporating the anticancer drug dacarbazine. The MN featured 700 μm high cylindrical bases and 300 μm conical tips, with diethyl fumarate added to improve mechanical properties. The study demonstrated a controlled drug release over 5 weeks, showcasing micro-stereolithography's potential in biomedical applications. However, the force required to penetrate the PPF/DEF and PPF/drug mixtures was significantly higher than typical skin insertion forces.^101^

Ali *et al.* demonstrated the advantages of DLP-based 3DP for fabricating PMNs using polyethylene glycol diacrylate (PEGDA). The method employed continuous vertical platform movement to achieve high surface quality. The study analyzed how polymerization time, light intensity, and resin composition affected production speed and geometric precision. Optimal MN had a length of 520 μm and a tip diameter of 40 μm, showing that DLP can produce biocompatible PMNs with precise dimensions and reliable performance, making it suitable for advanced biomedical applications.^102^ As several studies have mentioned, DLP-based 3DP has many advantages when fabricating polymeric MN.

DLP's layer-by-layer printing offers high accuracy and reproducibility, making it ideal for MN production on complex or curved surfaces. Lim *et al.* (2017) developed a dual-function, personalized MN splint using DLP 3DP for trigger finger treatment, combining MN-assisted TDD with splint immobilization. The MN splints, made from a commercial DLP projector and 3DM-Castable resin, could withstand forces twice that of an average thumb and enhanced diclofenac permeation through the skin within 0.5 hours. This study demonstrated DLP's potential for high-precision MN arrays on personalized, curved surfaces, surpassing earlier flat-surface designs.^103^ This dual approach could revolutionize trigger finger and related treatments. Research into modifying DLP resins for hydrogel-based PMNS, including optimizing photo initiators and crosslinkers, is ongoing. Zhou *et al.* (2024) developed a temperature-responsive NIPAM-AA-AM hydrogel MN using DLP, demonstrating drug delivery potential through temperature sensitivity.^42^

3D PMN patches with cone and pyramid geometries were made using Class 1 biocompatible resin, showing excellent mechanical strength and piercing ability. Soft lithography techniques, like DLP, were also employed to create master molds, with a “tanto blade” design to increase MN instability. The MN matrix was made from PVA and sucrose, while gold/silver nanocluster-labeled gelatin acted as a fluorescent probe for *in vitro* MN dissolution and skin behavior. The patch design was modified with four channels at the base to enhance MN separation post-insertion. This study highlights the potential for customizing MN designs and properties.^104^ In a similar study by Amer *et al.* (2020), two plant extracts, Vitex agnus-castus and *Tamarindus indica*, were delivered *via* PMN, which showed superior anti-cellulite effects compared to traditional MN devices and products. Six polymers were used, and the MN demonstrated similar efficacy in reducing inflammation and normalizing redox states in a guinea pig obesity model^105^

#### 3.2.3 Continuous liquid interface production (CLIP)

CLIP represents a significant advancement in 3DP technology and is considered to be the newest edition in the field of additive manufacturing developed in the year 2016.^13^ It offers an innovative fabrication method far different from the traditional layer-by-layer SLA methods, as it uses a specialized membrane that facilitates oxygen diffusion, preventing radical polymerization and enabling continuous printing. The process begins with a beam of UV light directed into a photopolymerizable liquid resin through an oxygen-permeable window. This window maintains a “dead zone” of non-polymerized, oxygen-inhibited resin above it, allowing for uninterrupted construction of the object rather than building it up layer by layer.^106^ The result is a technique that boasts high speed and impressive resolution. Despite its advantages, CLIP is currently expensive and not widely accessible or convenient for all users.

CLIP offers faster production of complex devices than SLA but faces challenges like limited biocompatible resins and poor mechanical properties. Advances in UV-curable materials are expanding possibilities, but they require thorough characterization and regulatory approval for clinical use. Drugs can be incorporated into CLIP-produced devices during or after production, with post-loading methods like drug absorption or surface coating preventing degradation but requiring additional steps. Bloomquist *et al.* (2018) developed a dissolving hydrogel PVA-based MN array for drug-loaded devices, investigating how geometry, crosslink density, and polymer composition affect the release of rhodamine-B (RhB) and clinically relevant drugs like dexamethasone-acetate and docetaxel. Their study confirmed CLIP's potential for controlled drug delivery.^85,106,107^ Different research groups who fabricated PMNs using CLIP and application with the materials recently have been listed out in Table 1. CLIP is a superior choice compared to SLA and DLP due to its continuous printing capability. Rajesh *et al.* demonstrated this by printing highly complex MN with unique designs that are difficult to replicate using material deposition, SLA, or DLP due to delamination issues. The square pyramidal MN features lattice structures made from various shapes, including triangles, tetrahedrals, and Voronoi patterns.^85^

**Table 1:** Overview of different polymeric 3D MN fabricated and its application based on VPP

VPP fabrication method	Materials used	Type and design of MN	Drug delivered/application	References
SLA	Biocompatible class I resin	Hydrogel hollow MN	TDD of rifampicin	10
		Flat pyramidal and spear-shaped	Insulin	113
		One plain bevelled tipped solid	Insulin	28
	Vinylpyrrolidone and PEGDA	Solid MN	Wrinkle management	103
	Poly(ethylene glycol) diacrylate (PEGDA)	Solid MN with a tapered design	Polymeric MN for transdermal insulin delivery	114
	Poly(lactic-*co*-glycolic acid) (PLGA)	Solid MN with conical tips	MN for vaccine antigen delivery	115
	Polyvinyl alcohol (PVA)	Hollow MN with internal channels	MN patches for controlled anti-inflammatory drug release	116
	Gelatin methacrylate (GelMA)	Solid MN with porous structure	DNA plasmids for gene therapy	61
	Methacrylate-based resin	Hollow MN for encapsulation	Doxorubicin/anticancer drug delivery	72
DLP	PVA and sucrose	Dissolving MN	Gold/silver nanoparticle loaded on to MN	102
	Polyurethane (PU)	Solid MN with conical tips	Vaccine antigens	45
	Poly(ethylene glycol) diacrylate (PEGDA)	Solid MN with micro-holes	Insulin	114
	Gelatin methacrylate (GelMA)	Solid MN with micropatterned surfaces	Contraceptive hormone in skin	34
	Polyvinyl alcohol (PVA)	Hollow MN for controlled release	Peptide hormone delivery like GH	40
	Poly(lactic-*co*-glycolic acid) (PLGA)	Hollow MN with micro reservoirs	DNA plasmids	26
	Polylactic acid (PLA)	Solid MN with sharp tips	Lidocaine	74
	Polycaprolactone (PCL)	Solid MN with micro-channels	Small molecule drugs (antihistamines)	82
CLIP	Silicon-based resin	Hollow MN with internal channels	Anti-inflammatory drugs/continuous release of therapeutics	50
	Poly(ethylene glycol) diacrylate (PEGDA)	Solid MN with micro-reservoirs	Insulin	87
	Poly(lactic-*co*-glycolic acid) (PLGA)	Hollow MN for encapsulating antigens	Vaccine antigens	90
	Gelatin methacrylate (GelMA)	Solid MN with micro-channels	Peptide hormones (*e.g.*, insulin-like growth factor)	35
	Polyurethane (PU)	Solid MN with sharp tips	Lidocaine	117
	Methacrylate-based resin	Hollow MN with encapsulation features	Melanoma	118
	Polyvinyl alcohol (PVA)	Hollow MN for controlled release	Doxorubicin/MN for anticancer drugs	74
TPP	Photopolymerizable resin (IP-Dip)	Micro-etched MN with a high aspect ratio	Contraceptive localized drug delivery	32
	Photopolymerizable acrylate resin (SU-8)	Solid MN with precise internal structures	DNA plasmids/gene therapy	110
	Poly(ethylene glycol) diacrylate (PEGDA)	Hollow MN with micro-channels	Insulin	119
	Gelatin methacrylate (GelMA)	Solid MN with optimized tips for antigen loading	Melanoma treatment	120
	Polyvinyl alcohol (PVA)	Solid MN with precise micro-patterns	Peptides (growth factors)	114
	Methacrylate-based resin	Hollow MN with controlled drug release features	Doxorubicin	13
	Polycaprolactone (PCL)	Solid MN with micro-channels for drug loading	Antimicrobial agents (antibiotics)	121

#### 3.2.4 Two-photon polymerization (TPP)

Multiphoton lithography, or TPP, is an advanced 3DP technology that utilizes rapid prototyping with various acrylate and methacrylate-based photosensitive resins and ultra-short laser pulses. TPP excels in creating intricate MN molds and solid MN arrays, achieving high precision through two-photon absorption from near-infrared femtosecond lasers, which simultaneously excite atoms to a higher energy state. This absorption creates a virtual state with higher energy, enabling precise polymerization at the laser's focal point. This targeted process minimizes absorption outside the focal area, allowing for the creation of intricate microscale and nanoscale structures suitable for commercial-scale production. TPP offers significant advantages in creating patient-specific devices and is suitable for clinical use in an energy- and cost-effective manner.^108^ Materials used in TPP must meet two criteria: they should absorb two photons simultaneously, either independently or with a PI, and be transparent to the laser wavelength. Suitable materials include SU-8 photoresist, methacryloxypropyl trimethoxysilane-zirconium propoxide copolymer, organically modified ceramics, ethoxylated trimethylolpropane triacrylate, polyethylene glycol diacrylate, and various acrylate- and methacrylate-based polymers.^109^ The materials used for TPP-based 3D fabrication of MN with application are listed in Table 1.

Compared to traditional MN fabrication techniques like reactive ion etching and lithography, TPP offers superior control over MN geometry and can be performed in standard clinical settings. In 2022, McKee *et al.* developed a solid MN array in a single step using TPP 3DP, screening various fabrication parameters and conducting simulations to study MN-skin interaction. This method improved resolution and specificity while reducing production time. It enables the creation of complex, fracture-resistant MN using low-cost photosensitive resins. Despite minor dimensional deviations and truncated tips, the MN was generally effective and comparable to control surfaces for human keratinocyte growth.^110^ In a study by Gittard *et al.*, the first group to use TPP for mold fabrication created MN from acrylate-based polymer with a diameter of 150 μm and a length of 500 μm, demonstrating good uniformity and durability. This prototype withstood axial loads up to 10 N, successfully penetrating the skin and creating open pores in the stratum corneum and epidermis for drug delivery.^99,111^

The commercialization of TPPs-based PMNs faces challenges such as ensuring batch-to-batch uniformity, refining designs for specific clinical applications, optimizing processing rates, and achieving cost competitiveness. Addressing these issues could improve the commercial viability of TPP in developing MN and other drug delivery devices.^112^ Currently, 2PP is limited to prototyping due to slow production speeds, hindering large-scale MN manufacturing. However, rapid replication molding using 2PP prototypes could enable the mass production of affordable MN patches for clinical use. In 2023, a 2PP master template was developed, eliminating the need for post-processing or harsh chemical treatments like silanization in PDMS mold production.^113^ The resin was added directly to the 2PP master template, followed by annealing, simplifying the PDMS mold system. This method allowed the reuse of the master template for drug delivery without further surface modifications.

## 4. Challenges of polymeric materials adapted vat photo-polymerisation (VPP) in pharma and medical applications

The development of 3DP PMN through the VPP technique holds promise for drug delivery but faces challenges. VPP technique offers superior fabrication advancement and is now considered a gold standard solution in printing MN for the transdermal application of drugs. Delving into the constraints of VPP technology, the polymerizable resins used in VPP often lack bioactive properties, limiting their clinical application. The resin must be non-toxic, biodegradable, and biomimetic for transdermal MN systems. Typical resins contain photoinitiators (PIs), stabilizers, and acrylates that can be toxic. Due to the rapid advancement and high-end research in this stream, adapting existing materials through polymer mixtures could meet clinical requirements, offering an alternative approach. Theoretically, using the right mixtures of polymers, any bio-compatible material can be used as a slurry resin in the open-source.^122^

**Fig. 11:**
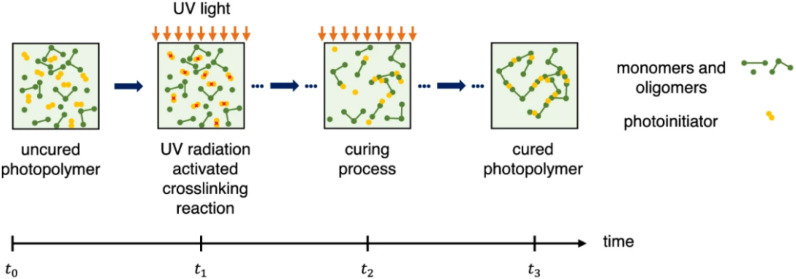
Schematic representation of the polymerization chain reaction steps (reproduced from A. Graca *et al.*,^123^ with permission from Elsevier B.V, © 2024).

VPP-based fabrication system for printing clinically relevant MN systems involves key components like multifunctional monomers, crosslinkers, plasticizers, pigments, and PIs. The monomers polymerize irreversibly, forming crosslinked networks through radical chain reactions initiated by UV light, with PIs catalyzing the process. PI absorbs the UV radiation emitted by the printer and finally harnesses the polymerization process. UV light, depending on wavelength and PI, generates free radicals that catalyze monomer–crosslinker reactions, forming stable, crosslinked networks and solidifying the resin. The polymerization chain reaction steps during the crosslinking are represented in Fig. 11. Resin slurries may also contain photo absorbers to enhance print resolution along with pigments which gives colours to the different patches. However, unreacted monomers, PIs, and crosslinkers can be toxic, and post-curing methods using ethanol or UV exposure may compromise biocompatibility and MN quality. For clinical use, VPP printing must address these challenges throughout the polymerization process.^123^ PI absorbs the UV radiation emitted by the printer and finally harnesses the polymerization process. UV light, depending on wavelength and PI, generates free radicals that catalyze monomer–crosslinker reactions, forming stable, crosslinked networks and solidifying the resin. Photoabsorbers in the resin enhance print resolution by controlling light penetration. The layer-by-layer method creates intricate designs based on the CAD file.^80^ Unreacted monomers, PIs, absorbers, and crosslinkers can be toxic. Post-curing with ethanol or UV exposure to remove unreacted groups may harm biocompatibility and affect the final MN patch quality. For clinical approval, direct MN printing for topical use must address all phases of vat polymerization.

**Fig. 12:**
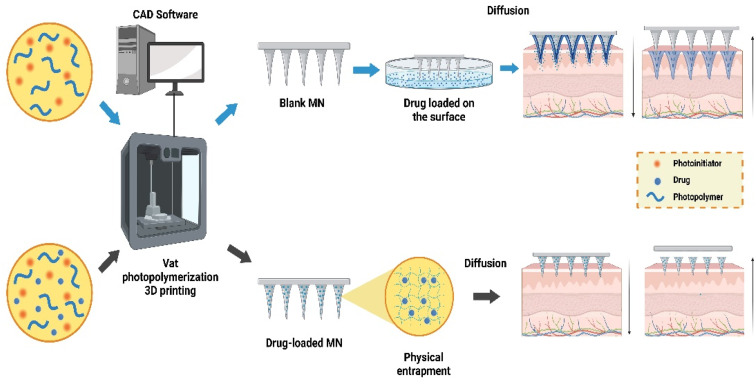
Schematic representation of the steps involved in the VPP 3DP for drug delivery mediated by MNImage created using BioRender (http://biorender.com/).

VPP 3DP is ideal for producing topical MN patches for TDD, offering precision, fast printing, strong bonding, and heat-free processing, which is suitable for heat-sensitive APIs. However, VPP resins are not typically GRAS-approved for clinical use. Developing biocompatible resin blends, such as PEGDMA, PEGDA, PCL, and PPF, could address this issue, though unreacted monomer residues require strict safety protocols for large-scale pharmaceutical use. Recent research has focused on developing custom resins for VPP technology by blending polymers with suitable PIs. The monomeric polymerization kinetics, mechanical properties, and VPP process parameters are key in selecting appropriate polymers. Methacrylated and epoxylated monomers are often used, and the type of PI significantly impacts printing accuracy, precision, and mechanical properties. PIs are categorized into type-1 (*e.g.*, TPO, BPO) and type-2 (*e.g.*, camphorquinone, benzophenone), with each affecting radical generation and light absorption. The combination PI, 1-phenyl-1,2-propanedione (PPD), uses both mechanisms, influencing color stability, accuracy, and mechanical performance. Type-1 PIs, like TPO and benzoyl peroxide (BPO), generate radicals through α-cleavage and absorb UV light. Type-2 PIs, such as camphorquinone, phenanthrenequinone, and benzophenone, work through hydrogen abstraction and absorb visible blue light (400–490 nm). The VPP process steps, including direct and indirect drug incorporation into PMNS, are shown in Fig. 12.^123,124^ The choice of PI affects the final product's colour stability, 3DP accuracy, conversion degree, and mechanical properties.^125^

This section reviews polymer types and techniques for creating lab-made resins, a relatively new area of research in 3DP transdermal applications. Customized resins for MN fabrication can save time and costs in drug delivery strategies. Goyanes *et al.* were pioneers in this field, using VPP (SLA) combined with FDM to create MN molds with APIs for anti-acne applications. They blended PEGDA and PED with TPO (PI) for polymerization, while PCL filaments loaded with salicylic acid were produced *via* FDM. SLA outperformed FDM in thermal stability and drug loading, highlighting SLA's advantages for producing high-resolution, flexible topical devices.^126,127^

Various technologies and customized resins have been explored to create personalized MN systems for specific patient needs. Caudill *et al.* used CLIP technology to fabricate MN patches for transdermal protein delivery, utilizing PEGDMA and TPO as PIs to produce a coating mask device with controlled protein loading.^127^ While MN fabricated using micro molds offered good precision, they suffered from long cycle times, mold shrinkage, and costly replication steps. A more efficient solution was to develop a single-step fabrication technique by optimizing a resin blend with polymer, PI, and APIs. Kundu *et al.* demonstrated this using PEGDA hydrogel with PI and diclofenac sodium, employing energy-exposed DLP to achieve high mechanical strength while retaining hydrogel properties. This method used pH and temperature stimuli for controlled drug delivery, producing intelligent hydrogel MN with optimized precision in one step.^128^ In 2019, Yao *et al.* evaluated key fabrication parameters for high-precision DLP printing of biocompatible MN for drug injection and retention. Using PEG400DA crosslinked under blue light with BAPO as the PI, this method enabled economical, single-step production of hydrogel MN, proving cost-effective and efficient for clinical applications.^129^

Reducing material use in thermoset resins for VPPs makes the technology more cost-effective and environmentally friendly. High-viscosity photopolymers limit efficiency, but lowering viscosity improves resin flow and printing speed. The optimal viscosity for SLA, DLP, and CLIP techniques is around 5 Pa s. Johnson *et al.* introduced low-viscosity resins using trimethylolpropane triacrylate and 2.5% TPO, enabling fast, versatile MN prototyping in various shapes and sizes in a single step. There have been many studies to improve the viscosity and limitations brought about by adding reactive diluents which interfere with the overall mechanical properties.^130,131^ Introducing dynamic covalent bonds in resins preserves their ambient performance by maintaining the polymer network, enhancing stiffness, rigidity, and elongation. Kuenstler *et al.* developed slightly crosslinked resins that maintained viscosity even with dynamic monomers in the SLA method. Low-viscosity resins reduced recoat time and minimized air bubbles during intricate MN printing, while highly viscous resins could slow down printing and negatively impact *z*-axis resolution.^132,133^

Biobased polymers like alginate, CMC-NA, oils, and proteins have been used as biocompatible additives in resins. Silk fibroin, for example, was added to poly(ethylene glycol)-tetraacrylate resins in the DLP system, enhancing the printing resolution of melanin nanoparticle-incorporated hydrogel MN. A library of biobased methacrylates with tunable mechanical properties was developed by Guit *et al.*, who also incorporated epoxidized soybean oil into photopolymer resins. A list of lab-made resins for high-resolution polymeric MN is shown in Table 2.^134^ A library of biobased methacrylates with tuneable mechanical properties was developed by Guit *et al.* This group also incorporated epoxidized soybean oil into the photopolymer resins.^135^ Single-step MN printing with biocompatible polymers loaded with APIs has been reported by various groups. Han *et al.* used μSLA to fabricate MN with backward-facing barbs for improved tissue adhesion, using PEGDA, BPO, and Sudan I as a photoabsorber. Gittard *et al.* employed Ormocer® b59 and PEGDMA, incorporating gentamicin sulfate to study antimicrobial properties. Yun *et al.* utilized μSLA and biodegradable PPF for precise MN fabrication. Lu *et al.* engineered drug-loaded propylene fumarate MN for transdermal chemotherapeutic delivery, adjusting viscosity with diethyl fumarate. These studies showcase advancements in MN technology, including biocompatible materials and enhanced functionality for specific medical uses.^89,136^

**Table 2:** List of lab-made resins customised to produce high-resolution PMNs

Photopolymer	Photo initiator	3DP technology	Drug/molecule	Possible applications	References
Poly(ethylene glycol) diacrylate (PEGDA)	Irgacure 2959	SLA	Insulin	Transdermal insulin delivery	63
Poly(lactic-*co*-glycolic acid) (PLGA)	2-Hydroxy-2-methylpropiophenone (HMPP)	DLP	Vaccine antigens	Vaccine delivery *via* MN patches	137
Gelatin methacrylate (GelMA)	Irgacure 2959	SLA/TPP	Peptide hormones	Controlled release of peptide hormones	59 and 138
Polyvinyl alcohol (PVA)	Camphorquinone (CQ)	DLP/TPP	Antimicrobial peptides	Antimicrobial peptide delivery	139
Polycaprolactone (PCL)	2-Hydroxy-2-methylpropiophenone (HMPP)	DLP/CLIP	Small molecules (*e.g.*, antihistamines)	Controlled release of small molecules	140
Poly(2-hydroxyethyl methacrylate) (pHEMA)	Irgacure 184	SLA	Peptides	Peptide drug delivery	64
Methacrylate-based resin	Camphorquinone (CQ)	SLA	Anticancer drugs	Anticancer drug delivery *via* MN	141
Poly(ethylene glycol) acrylate (PEG-A)	Irgacure 2959	SLA	Local anesthetics (*e.g.*, lidocaine)	Local anesthetic delivery	97
Poly(methyl methacrylate) (PMMA)	2-Hydroxy-2-methylpropiophenone (HMPP)	DLP	Hormones (*e.g.*, estradiol)	Hormone replacement therapy	137
Poly(urethane) (PU)	Irgacure 184	SLA	Antihistamines	Antihistamine delivery for allergic reactions	142

## 5. Physiochemical characterization of 3D printed PMN systems

A drug-loaded MN, either by coating or spraying, various physiochemical parameters are required to be analysed. Characterization of the morphology, dimension, mechanical strength, content uniformity, and physical and chemical stability are essential evaluation parameters.

### 5.1 Morphological, mechanical strength and stability

Evaluation of the morphology of PMNs is extensively carried out using scanning electron microscopy (SEM), often with gold coating for better imaging to clear off any electrical charges on the surface. Examination of the size, height, base, pitch, and additional essential physical parameters are obtained using SEM. Digital imaging of the needles using a range of magnifications between 30 to 120× has been reported to allow better resolution with detailed images of the MN. To ensure quick penetration and structural integrity of PMNS, they must be sharp and thin while maintaining drug concentration. Mechanical properties are tested using the Instron 5448 Micro Tester through axial and transverse fracture tests. Stability is assessed under varying temperatures and humidity, especially for sensitive drugs like vaccines or insulin, which may require stabilizers. Insertion force, proportional to the number of pores, is tested on neonate porcine epidermis and often with parafilm initially. Fracture resistance and flexibility are evaluated using a Texture Analyser. Lefkothea Antonara *et al.* (2022) found pyramid and conical MN optimal for skin penetration, with pyramids providing ease and cones offering mechanical strength. An ideal array (0.6 × 0.6 cm, 49 MN, 1 mm height, <75 μm tip diameter) achieved accurate printing and minimal pain. Chemical etching reduced MN dimensions, improving penetration and drug delivery. MN (2000–2500 μm length, 400–600 μm width, 170–220 μm tip) were reduced to 1–55 μm tips, emphasizing etching's role in effective drug delivery.^22^ Another study on 3D-printed MN (1200 μm height, 400 μm base, 50 μm tip) showed 97.2% skin penetration at 2.5 N, validated by Finite Element Analysis.^141^

A design optimization study compared Cone, Pyramid, and Spear MN (1000 μm) and found that Cone and Pyramid MN had higher fracture strength and required less insertion force than the Spear design, which was more prone to bending. The Cone design had slower drug release, while the Pyramid had uniform coatings and better drug release. Despite lower mechanical strength, the Spear design had the highest drug release efficiency. The safety index was highest for Pyramid, followed by Cone, and lowest for Spear. Another study with 1.2 mm MN showed tree-like MN had the highest strength and drug loading, while conical MN required the least insertion force, and spiral/ring-like MN balanced strength and drug release.^13,97^

### 5.2 Content uniformity and chemical composition

FDA guidelines require thorough evaluation of drug content and uniformity in MN. Achieving uniform coating is challenging, potentially affecting drug delivery. Drug homogeneity and coating uniformity are assessed using SEM, polarized microscopy, HPLC, and UV-visible spectroscopy. The dip coating technique ensures precise content uniformity, as seen in estradiol valerate MN. Chemical compatibility and stability are analyzed using DSC, TGA, and FTIR, with FTIR confirming polymerization stability in pyrrole MN and DSC revealing stability of estradiol valerate in almond oil and PEG400.^143^

### 5.3 Assessing toxicity: considerations in PMN system fabrication

Preliminary cytotoxic *in vitro* screening of PMN system follows ISO 10993-5 guidelines to evaluate biocompatibility and the effects of leached PIs or drugs. 3D-printed materials are submerged in cell media with human glioblastoma cells (U87-G cells), and confluency is assessed after 24 hours using microscopy.^144^ Similar tests have been conducted on human fibroblasts to assess cytotoxicity from MN leachable, and human embryonic kidney 293 cells, and bone marrow stromal cells have been tested using MTS assays to evaluate drug release. Drug release is assessed using porcine skin and the Franz diffusion system, with analysis by atomic absorption spectroscopy. A study on human HepG2 and U-87 MG cells showed biocompatibility of 3D-printed resin using the WST-8 assay, showing no significant growth difference from the control.^127^

Biocompatibility of 3D-printed PMN materials is crucial for safe application. Microfluidic resin used in additive manufacturing was tested on human dermal fibroblasts, showing 84 ± 3.55% viability for media-soaked disks and 68 ± 6.01% for UV-sterilized disks, compared to 97 ± 2.61% for controls. These results indicate generally acceptable cytocompatibility, with post-processing influencing cell response. This highlights the need to assess toxicity of 3D-printed PMN materials to ensure both functional performance and safety for biomedical use.^145^ Similarly, faceted MNs produced *via* CLIP enabled enhanced vaccine cargo coating and transdermal delivery in mice, eliciting robust humoral and cellular immune responses while relying on biocompatible 3D-printed materials.^146^

Several 3DP MN pharmaceuticals have undergone clinical assessments, highlighting the potential of this technology in personalized medicine. Spritam® (levetiracetam), an FDA-approved 3D-printed orodispersible tablet for epilepsy, is notable for its rapid disintegration and ease of administration. T21, for ulcerative colitis, utilizes targeted drug delivery with FDA approval of its investigational new drug (IND) application. FabRx's 3Dp chewable tablets for pediatric isoleucine delivery in maple syrup urine disease demonstrated effective control and patient acceptance. These examples showcase 3DP's impact on personalized therapeutics.

## 6. Biomedical and commercialisation aspects of 3D printed PMNS

Emerging nano-/micro-fabrication methods are advancing TDD systems, with MN offering reduced pain, less tissue damage, and precise API administration. 3D-printed MN technology represents a promising future for next-generation drug delivery and vaccination manufacturing. The pharmaceutical industry's diverse applications, including drug delivery, diagnostics, cosmetics, and inoculation, highlight the need for continued research to develop biodegradable polymeric systems that improve the efficiency of large, hydrophilic molecules.

Smart polymer MN for TDD offers controlled release by modifying polymer formulations, enabling targeted drug therapies. Biocompatible, biodegradable polymers ensure safety for internal use. 3D-printed PMN systems provide multifunctionality, supporting tissue fluid detection and a range of health applications, including clinical translation of pharmaceutical APIs. This technology promotes personalized drug delivery, such as sustained insulin release for diabetics and targeted cancer and wound healing therapies. It also finds applications in cosmetics, like anti-wrinkle peptide delivery. As demand for personalized, patient-compliant dosages grows, 3DP in TDD systems offers significant potential. However, challenges remain regarding toxicity, stability, and drug effectiveness, requiring further research to enable clinical applications. Despite all these advances, several challenges remain to be translated for clinical applications of polymer MN technology concerning the toxicity, stability, and effectiveness of drugs for use in fabrication. Further research on the solution to such problems will be directed so that this application can be widely used clinically.^75,147,148^

Industrial-scale production of PMN systems starts with selecting biocompatible, biodegradable materials like PLA, PLGA, and PVP, ensuring consistent quality for medical standards, and transitioning from laboratory experiments to large-scale manufacturing. Techniques such as hot embossing, injection molding, and micro-thermal forming are optimized, while automation boosts efficiency and reduces errors. Automation increases throughput and minimizes errors, enhancing production efficiency and consistency. CAD/CAM tools and 3DP aid precise design and prototyping, enabling rapid iterations. A pilot production line helps address scale-up challenges, ensuring a smooth transition to full-scale manufacturing and regulatory compliance.

Quality assurance in MN production involves Standard operating procedures (SOPs), real-time monitoring, and post-production testing to ensure consistent quality. Compliance with FDA and EMA guidelines requires biocompatibility, sterility testing, and adherence to good manufacturing practices (GMP). Packaging and sterilization, including gamma irradiation or ethylene oxide treatment, preserve MN integrity during storage and transport. Cost analysis estimates raw material, labor, equipment, and operational expenses, while scalability assessment identifies potential bottlenecks and inefficiencies to ensure successful large-scale production.^149^

Effective supply chain management is crucial for large-scale MN production, requiring strong supplier partnerships and efficient inventory systems. Market deployment includes clinical trials, regulatory approvals, and commercial launch strategies. Companies like 3M Drug Delivery Systems and Zosano Pharma have successfully scaled MN production, overcoming challenges in scalability, quality, and compliance. Polymeric MN, such as Zosano Pharma's ZP-Glucagon for hypoglycemia and Micron Biomedical's vaccine patches, demonstrate their potential for drug delivery and improved patient compliance. Vaxart's MN patches enhance oral vaccine delivery, and 3M and West Pharmaceutical Services have developed various MN products for drug delivery and vaccination. In conclusion, successful MN production requires careful planning across materials, manufacturing, compliance, and supply chain to enable effective clinical and commercial deployment.

## 7. Ethical consideration of 3-D printed PMNS

The development of a 3DP PMN system for TDD raises ethical concerns around safety, accessibility, patient autonomy, data privacy, regulatory compliance, and environmental impact. Ethical bodies like the World Medical Association (WMA) and UNESCO oversee responsible innovation, addressing dual-use concerns to prevent misuse. While advancing medical technology is crucial, it must balance with ethical considerations to protect patient safety, privacy, and equity. These bodies offer guidelines to navigate these issues responsibly.^150^ Ethical considerations for 3DP PMN include rigorous safety testing, equitable access, and patient autonomy. Informed consent, data privacy, and regulatory oversight are essential to maintaining trust and ensuring safety. Environmental impacts should be addressed through sustainable practices. Ethical research conduct, including animal and human testing, is vital, and innovation must be balanced with patient safety and fairness. Ultimately, responsible advancement is key to the success of this technology.

### 7.1 Regulatory clearance and clinical safety

Theranostics has become essential for evaluating the clinical relevance and translation of drug delivery systems. Regulatory approval and quality control remain key barriers to ensuring public health protection. MN applications are relatively new in pharmaceuticals, with FDA guidelines for 3D-printed medical devices highlighting challenges in standardization due to varying materials and process parameters. In 2020, the FDA issued guidance on safe data sheets for 3D-printed MN, addressing risks like skin infections, nerve damage, scarring, and allergic reactions. Sterilization remains a concern, as methods like gamma radiation and ethylene oxide may harm devices or formulations. While advancements in sterilization-capable 3D printers are possible, FDA guidelines must be followed. Experts believe MN's architectural variety offers promising applications in pharmaceuticals and therapy.^78,148^ An expert opinion stated that MN, with various options with architecture, allows research for application in the pharmaceutical sciences and therapy.

### 7.2 Safety and efficacy

Ensuring patient safety is crucial. 3D-printed MN must undergo rigorous testing to prevent adverse reactions like skin irritation, infection, or toxicity. Clinical trials require approval from ethical bodies such as IRBs to prioritize safety. Long-term effects, especially for chronic conditions, must be thoroughly studied, as continuous use may lead to cumulative side effects that should be disclosed to patients and healthcare providers. A regulatory body, such as the United States Food and Drug Administration (FDA) and the European Medicines Agency (EMA), will ensure that all these questions are adequately addressed before being approved for market access ethical issues also arise with animal testing, overseen by organizations like IACUC, which ensures humane treatment and the use of alternatives when possible. In human trials, participants must be fully informed and consent voluntarily.^151^ There is a need to study the long-term effects of the usage of 3D-printed MN fully, especially in the case of chronic conditions. Continuous usage, therefore, can result in cumulative side effects that ought to be pointed out to patients and healthcare providers. When advancing to human trials, participants must be fully aware of the experimental nature of the technology and consent voluntarily.^152^

### 7.3 Patient autonomy and informed consent

Advanced 3DP technology may not be accessible to all, especially low-resource populations. Organizations like the WHO and national health ministry's advocate for fair technology distribution to ensure equal benefits. Economically disadvantaged patients may be excluded due to high development and deployment costs, prompting calls from ethical bodies like the NIH to create affordable solutions. Patients must be fully informed about the benefits, risks, and experimental nature of 3D-printed MN. Informed consent is overseen by ethical bodies like IRBs to protect patient autonomy. While self-administration can empower patients, it raises ethical concerns like misuse, incorrect dosing, and lack of medical supervision, requiring careful oversight and patient education.^82^ Ethical oversight has to ensure that the protocols for self-administration contain adequate patient education and safeguards.^153^

### 7.4 Data privacy and security

The use of smart MN systems for health monitoring raises privacy and security concerns regarding patient data. Regulations like the General Data Protection Regulation (GDPR) in Europe and the Health Insurance Portability and Accountability Act (HIPAA) in the U.S., impose strict data protection rules, ensuring patients are informed about the data collected and its usage. Transparency in data practices fosters trust between patients and healthcare providers. Additionally, ethical concerns arise when devices lack regulatory oversight. Agencies like the FDA and EMA must ensure 3D-printed MN meet safety and efficacy standards, with clear accountability for device failures, ensuring manufacturers, healthcare providers, and regulatory bodies uphold safety.^154^

### 7.5 Environmental impact

The production and disposal of 3D-printed MN and associated materials raise environmental concerns. Ethical bodies, including environmental protection agencies and sustainability organizations, advocate for minimizing waste and using sustainable materials in the development of these technologies.^81^ If non-biodegradable materials are used, there could be long-term environmental consequences. Ethical oversight encourages the development of biodegradable and environmentally friendly MN systems.^14^

## 8. Challenges and future potentials

The quest for biodegradable and biocompatible MN is gaining momentum in the field of TDD, especially in clinical and pharmaceutical applications. However, challenges remain before their widespread use, including skin irritation, immune responses, micro-level contamination, mechanical weaknesses, and issues with drug loading and delivery of hydrophilic macromolecules. The emergence of 3-D printed PMN systems offers promise as a customizable, nanotechnology-based drug delivery system. 3-DP, especially the VPP method, enables precise fabrication of drug-laden MN in a short time. Despite its potential, challenges persist, such as the removal of unreacted monomers, drug–polymer interactions, temperature effects, optimization of printing parameters, and regulatory concerns. Nevertheless, ongoing research aims to overcome these obstacles, paving the way for the commercialization of 3DP PMN for large-scale transdermal delivery.^34^ Integration of compatible polymers in resins of 3D printers to fabricate MN for clinical uses has brought about a digital transformation in reshaping drug delivery systems. Even with these drawbacks, the research attempts to optimize and use the 3DP PMN system as a substitute for hypodermic needle-based drug delivery is considered for commercialization on a large scale.

Beyond 3DP, 4D printing represents an advanced technology in pharmaceuticals, enabling programmed shape deformation in response to external stimuli. This innovation could enhance drug delivery, particularly as researchers work to improve MN adhesion to the skin. Combining 3D-derived MN with microfluidic systems and specialized reservoirs for controlled drug release holds promise for future applications. Future research may lead to a universal polymer resin compatible with all VPP-based 3DP systems, simplifying process optimization and reducing variability. By minimizing crosslinkers, photopolymers, and PIs in the resin, it could improve printing precision, enhance mechanical properties, and reduce toxicity, advancing towards clinical-grade systems.

With proper guidelines and regulatory approval, 3DP PMN systems have vast potential in pharmaceutical applications. The key challenge for VPP technology is achieving the same quality, repeatability, and production scale as existing pharma products. While PMN systems can deliver a wide range of drugs transdermally, they still fall short in comparison to market standards. Future efforts should focus on overcoming these challenges to enable large-scale commercialization of 3DP PMNS. These MN could revolutionize drug delivery, offering a convenient alternative to oral and hypodermic methods. As 3D and 4D printing technology advances, smart devices and TDD systems with proper ethical clearance are expected to become a widely marketed reality.

## 9. Conclusions

Recent advancements in MN drug delivery systems over the past decade have revolutionized pharmaceutical applications, offering advantages in cases where traditional methods fall short or cause discomfort. Polymers, known for their biocompatibility, are the most widely used materials in MN manufacturing, especially in 3DP technologies. Polymeric MN offers distinct benefits over silicon and metal types, driving their increasing relevance and promising future. This review highlights drug delivery technologies using micro-molding and additive manufacturing, with a focus on custom-made resins to address toxicity challenges. Key topics include the physiochemical properties, biomedical applications, and regulatory and ethical concerns of self-dissolvable PMNS. The review summarizes recent progress and challenges in 3DP PMNS, which could streamline TDD. Ultimately, personalized drug delivery through commercially available 3D-printed PMNS, due to their convenience and patient compliance, has the potential to be a game changer, contributing to improved living standards in the era of smart devices.

## Author contributions

Geethu Madhusoodanan: writing – original draft, writing – review and editing, data curation, conceptualization; Amrita Arup Roy: writing – original draft, data curation, writing – review and editing; Tejaswini Kalkundri: writing – review and editing, data curation; Namitha K Preman: writing – review and editing, data curation; Deepanjan Datta: writing – review and editing, data curation; Namdev Dhas: writing – review and editing, data curation; Komal Rana: writing – review and editing, data curation; Srinivas Mutalik: writing – review and editing, conceptualization, supervision, formal analysis, validation, resources.

## Conflicts of interest

The authors declare no competing interests. The views and opinions expressed in this article are purely those of the authors.

## Data Availability

No primary research results, software or code have been included and no new data were generated or analysed as part of this review.
